# Protecting Newborns from Multidrug-Resistant Infections: The Emerging Role of Bacteriophages

**DOI:** 10.3390/v18060664

**Published:** 2026-06-12

**Authors:** Olaf Bajrak, Andrzej Górski, Ewa Jończyk-Matysiak

**Affiliations:** 1 Bacteriophage Laboratory, Hirszfeld Institute of Immunology and Experimental Therapy, Polish Academy of Sciences (HIIET PAS), 53-114 Wrocław, Poland; olaf.bajrak@hirszfeld.pl (O.B.); andrzej.gorski@hirszfeld.pl (A.G.); 2 Phage Therapy Unit, Hirszfeld Institute of Immunology and Experimental Therapy, Polish Academy of Sciences (HIIET PAS), 53-114 Wrocław, Poland; 3 Department of Clinical Immunology, Medical University of Warsaw, 02-006 Warsaw, Poland

**Keywords:** ESKAPE group, newborn infections, infant microbiota, phage therapy

## Abstract

Newborns may suffer from dangerous bacterial infections caused by life-threatening multi-drug resistant pathogens. Thus, despite bactericidal capabilities of antibiotics, microorganisms are known to circumvent this therapy, and a new, more effective type of remedy is needed. An increasingly recognized strategy for addressing these challenges is the use of bacteriophages—viruses infecting bacteria—collectively referred to as phage therapy. Nonetheless, the research considering phage therapy amongst newborns is still at a pioneering stage, owing to the scarcity of systematic investigations and the prevalence of case-study data, leaving room for further discovery and analysis. This review summarizes the information needed to understand this complex issue, considering the description of pathogens causing infections affecting newborns, the formation of the early microbiota and phageome (defining its composition followed by an influence on immune system development), and the possible use of bacteriophages in the treatment, which may be complicated by ethical concerns.

## 1. Introduction: Core Bacterial Species in Terms of Forming a Microbiota and Their Potential Pathogenicity

A continuing debate concerns whether the neonatal microbiota may begin to form in utero. Several studies have reported the presence of *Proteobacteria* and *Enterobacteriaceae* in the placenta, amniotic fluid, and meconium [1,2,3], suggesting a possible maternal–fetal microbial transfer. However, an equally substantial body of evidence emphasizes that contamination cannot be fully ruled out in these cases and maintains that the womb is sterile [4,5,6]. Although there is still no definitive proof of a true prenatal microbial transmission, it is agreed that the first months of gut microbiota development are crucial for adulthood and affect the formation of an immune system [7,8]. Hence, if it does not proceed correctly, various abnormalities, such as the development of asthma [9], and more severe consequences, including multi-sensitized atopy and altered T-cell differentiation [10], and growth impairment (stunted children [11]) or increasing risk of celiac disease and type I diabetes [12], can occur. Commensal bacteria also take an important part in the education of the immune system by presenting foreign antigens [13]. Furthermore, the first bacteria colonizing an infant’s intestinal tract influence further neurological development and gastrointestinal metabolism [14]. This process, which determines microbiota composition, depends on multiple factors, including the mode of delivery, breastfeeding practices, maternal health, and antibiotic use. Among these, the first two are considered the most significant, as they largely determine the diversity and dynamics of the early microbiota [15,16,17]. Postnatal microbiota initially consists of both obligate and facultative anaerobic bacteria, including *Enterococcus*, *Lactobacillus*, *Bacteroides*, *Enterobacter*, and *Staphylococcus*, which are considered to be fundamental early colonizer microorganisms shaping the infant gut microbiota, and consequently, affecting the development of the immune system [18,19].

As mentioned, one of the key factors determining the composition of the gut microbiota is the mode of delivery, which plays a crucial role in its proper development [20]. It has been shown that neonates born vaginally predominantly acquire microorganisms colonizing the maternal vagina, including *Prevotella*, *Lactobacillus*, or *Sneathia* spp. Moreover, they may also harbor other bacteria, e.g., *Clostridium innocuum*, *Bacteroides xylanisolvens*, *Phocaeicola vulgatus*, as well as potentially pathogenic species such as *Escherichia coli* and *Bacteroides thetaiotaomicron* [21,22]. In contrast, newborns delivered by caesarean section (C-section) predominantly inherit bacteria resembling maternal skin microbiota rather than vaginal microbiota. These include *Corynebacterium*, *Staphylococcus* (e.g., *Staphylococcus aureus*; *Staphylococcus hominis*), *Propionibacterium*, *Klebsiella*, *Enterobacter*, *Clostridium perfringens*, and *Veillonella* (e.g., *Veillonella dispar*; *Veillonella infantium*; *Veillonella parvula*) [20,21,22]. Furthermore, gut microbiota colonization in cesarean-delivered infants is typically delayed as a result of the absence of naturally occurring vaginal symbionts (e.g., *Bifidobacterium*, *Lactobacillus*, *Bacteroides*, *Parabacteroides),* leading to an increased abundance of opportunistic pathogens and heightened susceptibility to infection caused by an immaturity of the neonatal immune system and gut barrier [20,23,24,25].

**Figure 1:**
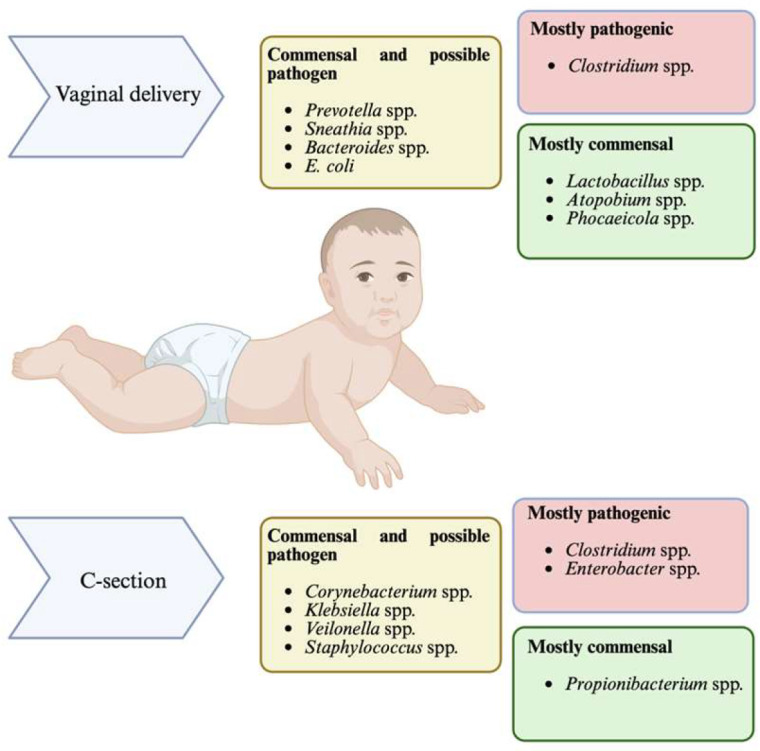
The most relevant bacteria involved in forming an infant gut microbiota divided between specific delivery modes. Created in BioRender. https://BioRender.com/fj9f0mn.

Moreover, children born via C-section experience a disrupted mother-to-newborn transmission of microorganisms, which may suffer from heightened risk of obesity, asthma, type-1 diabetes, or celiac disease [12]. The most relevant bacteria associated with specific delivery modes are summarized in Table 1 and Table 2, and Figure 1.

**Figure 2:**
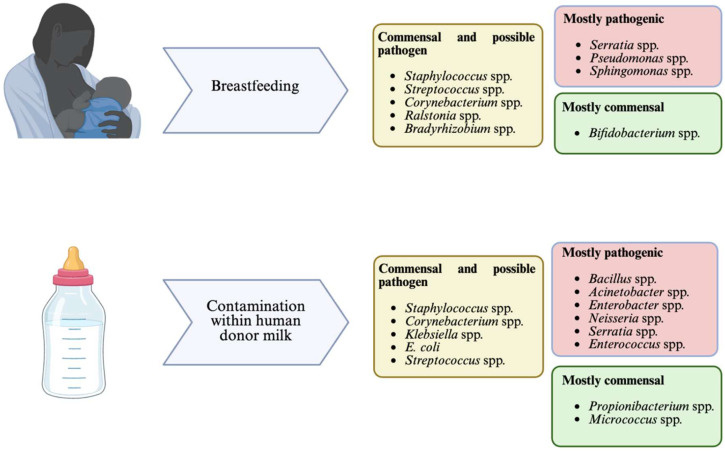
The most relevant bacteria involved in forming an infant gut microbiota divided between natural breastfeeding and possible contaminations of human donor milk. Created in BioRender. https://BioRender.com/fj9f0mn.

Furthermore, an infant’s gut microbiota composition depends on type of feeding [15,168]. It is well established that breast milk is a complex biological fluid rich in nutrients essential for a proper infant development. These components can be broadly classified into two groups: macronutrients (e.g., lactose, whey, fatty acids, caseins and HMOs) and micronutrients (e.g., immunoglobulins classes G, M and A, calcium and vitamins) [169,170,171,172,173,174,175,176,177,178,179]. Despite the importance of these factors in terms of an early development, the composition of human breastmilk microbiota represents one of the primary drivers of infant’s microbiota formation [180]. Nevertheless, not only the bacteria present in breastmilk but also mother’s skin microbiota contribute significantly to the arrangement of infant’s microbiota. However, in the context of breastfeeding, these two sources of microorganisms are generally referred to as the “breastmilk microbiota”. It is dominated mostly by species belonging to the genera *Staphylococcus*, *Serratia*, *Streptococcus*, *Pseudomonas*, *Corynebacterium*, *Ralstonia*, *Propionibacterium*, *Sphingomonas*, *Bifidobacterium*, and *Bradyrhizobiaceae* [181,182]. The occurrence of various bacteria in the breastmilk including both commensal and potential pathogens may suggest that the development of a synthetic or donor-derived milk preparation devoid of bacterial pathogens could represent a safer alternative. Nonetheless, donor human milk is often contaminated with potentially harmful bacteria, such as methicillin-resistant *Staphylococcus aureus* (MRSA), *S. epidermidis*, *Bacillus cereus*; *Bacillus anthracis*, *Corynebacterium tuberculostearicum*, *Enterobacter cloacae*, *Klebsiella oxytoca*; *Klebsiella pneumoniae*, *E. coli*, *Neisseria elongata*, *Streptococcus mitis*; *Streptococcus oralis* [183]. Despite this, bacteria present in human milk play a vital role in maintaining mucosal immune function and regulating cytokine activity within the enteric nervous system [184]. The most relevant microorganisms associated with breastfeeding and possible contaminations are summarized in Table 3 and Table 4, and Figure 2.

As mentioned, maternal health and antibiotic exposure are also important factors contributing to the formation of the infant gut microbiota [15,22]. Many conditions affecting maternal microbiota, e.g., maternal diet, lactation, infections (which might lead to antibiotic treatment), and other health-related factors, influence the microbial communities transmitted to the newborn [15]. Thus, both maternal health condition and antibiotic exposure have been associated with delayed microbiota maturation, susceptibility to opportunistic pathogens, and decreased microbial richness and/or diversity [15,18]. Furthermore, exposure to antibiotics during pregnancy, delivery, or early postnatal life can be correlated with an extensive resistome within bacteria residing in an infant’s gut [18]. While these factors undoubtedly contribute to shaping the neonatal microbiome, their effects are often highly individualized. As a result, their impact on microbial colonization is more difficult to generalize across populations. In contrast, delivery mode and infant feeding practices represent more predictable determinants; hence, these factors have been more extensively investigated and are discussed in greater detail throughout this review.

## 2. Forming a Gut Phageome

Bacteriophages are amongst the earliest colonizers of newborns microbiota [270]. It has been estimated that tailed phages constitute a vast majority (95%) of viral particles present in human milk and infant stool [271]. Although most of these “pioneer” phages are thought to be double-stranded DNA (dsDNA) temperate phages, induced from bacterial strains transmitted from mother to child [272,273,274,275,276], this pattern shifts later in life, when lytic phages become more predominant [277]. It is well established that the neonatal gut at birth is free of phages and becomes rapidly colonized during the first days of life [278]. The most important transmission routes include human milk, delivery mode [279], close maternal contact, environmental exposure, and oral intake [271]. Although the core phageome of preterm human donor milk consists mostly of phages with a siphovirus type of morphology infecting *Staphylococcus*, *Propionibacterium*, *Enterobacter*/*Klebsiella*, *Escherichia*, *Pseudomonas*, and *Enterococcus* [276], the human gut phageome might be, in later stages, dominated by CrAssphages (cross-assembly phages, mostly exhibiting podovirus morphology) infecting *Bacteroidetes* and *Prevotella* spp. [280,281]. This underscores the importance of multiple factors shaping the phageome during early life. Nevertheless, another study demonstrated that the commonly recognized CrAssphages were outnumbered by phages infecting *Clostridiales* and *Bifidobacterium* [282]. Furthermore, in a comprehensive analysis performed by Rybicka & Kaźmierczak, it was proven that the group of phages colonizing an adult’s gut microbiota consists mostly of phages exhibiting a host range involving *Actinobacteria*, *Bacteroidetes*, *Firmicutes*, and *Proteobacteria*, with a prevalence of phages with a siphovirus type of morphology [283]. This metagenomic analysis supported not only the fact, which was proven by Li et al., that phageome is highly dynamic throughout the first years of life and highly individualized [284], it also has shown that phageome is diverse across different body niches [283,285].

Since bacteriophages are viruses that infect and kill bacteria, the infant’s phageome is thought to a major determinant of the formation of gut microbiota during the first years of life by influencing bacterial ecology [271]. Moreover, the immature infant’s immune system is indirectly supported by bacteriophages that maintain bacterial populations, simultaneously limiting pathogen colonization [286]. For instance, through a predator–prey relationship, bifidophages—phages infecting bifidobacteria and transmitted via human breast milk—may alter the bifidobacterial population [287], a pattern similar to that observed in terms of phages infecting *S. epidermidis* [143]. Alterations in gut phageome have been associated with several disorders, including inflammatory bowel disease, type I diabetes, and colorectal cancer [271], and have even been proposed to correlate with NEC [288]. Nevertheless, these abnormalities may derive from a sunken bacterial diversity, which could be a reason for the domination of dsDNA tailed phages (previously classified as *Caudovirales*) regarding phages formerly classified as *Microviridae*. These ratios are challenging to determine via monitored phage therapy, since used phages are intended to infect specific bacterial pathogens [281,289].

Although the phageome is highly dependent on the overall microbiota composition, Leal Rodríguez et al. revealed that phages solely may increase the risk of child preschool asthma [290]. Conversely, lower phage concentrations in the nasopharyngeal area may also contribute to asthma [291]. Moreover, several studies have shown that the abundance of *Propionibacterium* spp., modulated by *Propionibacterium*-specific phages in the respiratory virome, is correlated with recurrent respiratory tract infections and elevated serum cytokines, suggesting that bacteriophages may influence the immune system [292]. The immune development in early life depends on the maternal immune system, autoantigens, and microbiota composition, including both pathogens and commensals [293]. However, bacteriophages are also involved in the development of an infant’s immunity [294], as they are known to induce both pro- and anti-inflammatory immune responses [295,296,297,298]. Nevertheless, it varies depending on the phage, host pathogen, and the condition of immune system itself (e.g., immune competence or inflammatory state). This variability represents one of the main challenges that scientists must address to establish phage therapy as a standardized, non-experimental medical approach, applicable not only in terms of treating newborns but also adults. For instance, it was proven that T4 phage can be trafficked inside the human cell in order to prevent triggering the immune system by being internalized in macropinosome, making its genome unavailable for TLR9 and cGAS-STING pathways [299]. This suggested that mammalian cells might harvest phages in order to enhance their metabolic activity; nevertheless, there is no data regarding this mode of action regarding an immature immune system. Interestingly, phage PM16 was proven to enhance long-term immunity against its host (*Proteus mirabilis*) caused by macrophage priming [300]. On the other hand, Pf phage (infecting *P. aeruginosa*) not only produced its mRNA within eukaryotic cells but also suppressed bacterial clearance via triggering antiviral immune response [301]. Importantly, phages can act as a natural barrier supporting the immunity. For instance, they are known to adhere to the mucosal layer, which may interfere with bacterial attachment and penetration [281]. Furthermore, as a result of transcytosis, phages are able to penetrate these layers and circulate in the blood [296]. In terms of adults, the immune systems can remove bacteriophages via mononuclear phagocytes, especially those present in the liver and the spleen, the major organs responsible for phagocytosis of circulating bacteriophages. Importantly, phages can also induce the production of specific IgM antibodies, which may subsequently undergo a class switching to IgG and IgA, as they are recognized by the human immune system as foreign biological entities [302,303]. Taking into account the immaturity of a newborn’s immune system, it is crucial to enhance research in that field, before defining phages as safe antimicrobial agents in terms of treating newborns infected with multidrug-resistant (MDR) pathogens.

## 3. Antibiotic Resistance Among Bacterial Pathogens Affecting Newborns

Antibiotic resistance among bacterial pathogens became one of the biggest threats, especially to immunocompromised patients, both elderly and newborns [268,304]. In 2017, WHO published a list of bacterial pathogens that required urgent action in terms of seeking an alternative for an antibiotic treatment—the ESKAPE group [305]. It refers to bacteria that currently pose a major global threat because of their genomes carrying genes encoding antibiotic resistance mechanisms: *Enterococcus* spp.; *S. aureus*; *K. pneumoniae*; *A. baumannii*; *P. aeruginosa*; *Enterobacter* spp. [306]. It was estimated that antimicrobial resistance within bacterial communities leads to 700,000 deaths annually, of which 200,000 are newborns [307]. Many neonatal intensive care units (NICUs) report an increased abundance of drug-resistant pathogens isolated from patients every year, many of which are resistant to commonly used antibiotics in these facilities [268,308,309,310,311]. This issue affects newborns not only directly but also indirectly through infected pregnant women carrying MDR bacterial strains, which may be vertically transmitted to their children [312,313]. Furthermore, one of the major sources of bacterial strains can be stretcher mattresses, humidicrib mattress fillings, and newborn feeding bottles, which can be colonized with bacterial biofilm (a structure that enhances bacterial survival and resistance to antibiotics and conventional disinfectants) [314,315,316]. Moreover, biofilm-forming bacteria can also be localized within endotracheal tubes used for a mechanical ventilation, which may lead to serious respiratory tract infections such as ventilator-associated pneumonia (VAP) [317]. Furthermore, children staying in the NICU can also be exposed to MDR pathogens through toys, which are often colonized by pathogenic bacteria, e.g., *S. aureus* and *A. baumannii* [318,319]. There have already been reported cases of newborn deaths caused by MDR pathogens such as *K. pneumoniae*; *Staphylococcus* spp.; *E. faecium*; *Streptococcus* spp.; *E. coli*; *E. cloacae*; *A. baumannii* [263,311,320,321,322,323]. In terms of neonates infected with pathogenic bacteria, timely therapeutic intervention is vital. However, whenever the identification of an effective antibiotic is necessary, the initiation of appropriate treatment is inevitably delayed, thereby prolonging the overall therapeutic process. It has been calculated that, regarding bloodstream infections, each hour of delay increases the mortality rate amongst children by 7.6% [324]. Worldwide, sepsis is estimated to occur approximately 3 million times per year in NICUs, with a mortality rate of 11–19% [325]. Moreover, these numbers increased by over 2% for 14th to 21st day of treatment in China [326]. Considering that neonatal infections are one of the greatest threats to newborn lives (mortality rate between 7% and 14%, estimated to claim 2 to 9 million children annually [327]), and since all bacterial species listed in Table 1, Table 2, Table 3 and Table 4 have been reported to resist many antibiotics [220,328,329,330,331,332,333,334,335,336,337,338,339,340,341,342,343,344,345,346], there is an urgent need to seek alternative treatment options for bacterial infections that can more effectively protect vulnerable early-life patients.

## 4. Bacteriophages vs. Antibiotics

Bacteriophages (phages) are now considered as an alternative for combating infections caused by MDR bacterial pathogens [289,347]. Phages are viruses that infect bacterial cells, amplify within them and lyse (kill) the host from the inside [348]. The most common types of bacteriophages are lytic phages (undergoing lytic cycles) and temperate phages (undergoing lysogenic cycles). The lytic cycle involves an adsorption of phage receptor binding proteins (RBPs) to receptors on the bacterial cell surface, a replication of the phage genome, an assembly of new virions within the bacterial cell and a lysis of the host cell, thereby completing the cycle. These phages are recommended for therapeutic use. Temperate phages, after the adsorption and injection of their nucleic acid, can integrate with the bacterial genome, stay dormant as prophages and enter the lytic cycle whenever specific environmental conditions occur, lysing their host cell from within [349]. Phage therapy involves an administration of phages to infected patients in order to eradicate pathogenic bacteria. Despite the fact that temperate phages may enhance the effectiveness of antibiotic treatment through phage–antibiotic synergy [350], they are not considered suitable for a therapeutic use, as a result of their ability to acquire bacterial genes (e.g., encoding bacterial toxins and/or antibiotic resistance) and spread them within bacterial communities [351]. Lytic phages, however, are regarded as one of the most promising alternatives for treating infections caused by MDR pathogens, as a successful treatment of every representative from the dangerous ESKAPE group was performed [315]. Despite their capacity to adapt to their host, through an evolution process [352], phages generally exhibit high specificity to their bacterial host. Hence, bacteriophages in the neonatal setting could be used as a targeted intervention against high-risk bacterial colonization. In selected high-risk populations, particularly preterm infants and patients hospitalized in NICUs, waiting until the particular symptoms occur (e.g., sepsis) may limit the opportunity for a successful treatment. Unlike broad-spectrum antibiotics, phages can provide this effect while leaving commensal bacteria “untouched”, hence protecting neonates with the disruption of their microbiota during its critical, developmental stage. Although further clinical studies are required, this targeted approach may represent a promising strategy for reducing the risk of infection associated with colonization by MDR pathogens.

As mentioned, shaping the gut microbiota of a newborn in the first months of life is crucial for a proper immunological and/or neurological development [10,14]. Therefore, antibiotic treatment, which is often prolonged in cases involving MDR pathogens, and reduces the diversity of microbiota [353], may lead to developmental disadvantages. These induce an increased risk of asthma, food allergies, obesity, diabetes, inflammatory bowel disease, psoriasis, developmental delays in motor skills, communication abilities, cognitive functions, and even autoimmune diseases [354,355,356,357,358,359,360,361,362,363]. For instance, intrapartum antibiotic use has been associated with a reduced abundance of lactobacilli and bifidobacteria in neonatal stool [12]. Furthermore, antibiotic administration not only disrupts the equilibrium of an infant’s microbiota but may also increase the abundance of pathogenic bacteria or even elevate the risk of NEC. This highlights that antibiotics can act as a double-edged sword, especially in the management of preterm infants [364,365]. Gudnadottir et al. in 2025 also demonstrated that antibiotics used during the early stages of childhood may increase the risk of epilepsy [366]. Nevertheless, an appropriate probiotic supplementation following the antibiotic treatment may mitigate the aforementioned adverse effects of an antibiotic exposure in the postnatal period [367]. It has been shown that the likelihood of developing a resistome (defined as the composition of all antimicrobial resistance genes present among bacterial strains within the population [368]) in the infant organism, as a consequence of antibiotics exposure during the neonatal period, is low [369]. Nevertheless, some studies have shown that the spread (through horizontal gene transfer) and persistence of resistance genes within the gastrointestinal microbial community can be long-lasting, following the antibiotic treatment administered in the postnatal period [370,371,372]. In conclusion, although phage therapy represents a promising alternative to antibiotics in the treatment of neonatal infections, further well-designed studies are required to fully assess its efficacy and safety, particularly with regard to the complexity of interactions occurring between bacteriophages and the immune development.

## 5. Phage Application to Cure Infections Caused by Neonatal Pathogens

Since the emergence of MDR pathogens, phage therapy has increasingly become one of the few viable alternatives for treating bacterial infections [289]. Phages infecting most bacterial species, listed in Table 1, Table 2, Table 3 and Table 4, have already been characterized in vitro [373,374,375,376,377,378,379,380,381,382,383,384,385,386,387,388,389,390,391,392]. The number of case studies on personalized phage therapy continues to increase annually; however, only a few reports describe treatments involving pediatric patients [393]. Although studies on the use of phage therapy during pregnancy remain scarce, in terms of pregnant and perinatal periods of phage therapy, there have been proposed key aspects of a proper therapy protocol, which might be also applicable in terms of the postnatal period [394]. Firstly, strictly lytic phages should be considered as appropriate antimicrobial agents, as a result of their inability to integrate within bacterial host genetic material and to spread potentially unfavorable genes within bacterial communities. Furthermore, it is essential to comprehensively understand the infection that is to be treated. Thus, the selective elimination of a dominant pathogen may inadvertently create ecological space for secondary, less abundant microorganisms to proliferate and initiate a subsequent infection. Furthermore, there is an obstacle to be addressed regarding the optimal dose of administered bacteriophages during the therapy, as well as proper clinical monitoring [395]. Nevertheless, it was calculated that 91% of case studies involving phage therapy were successful with slight side effects, which stands for a great opportunity in terms of the treatment of neonates from infections caused by MDR pathogens [396].

In 1999, one of the first documented successful treatments of a newborn was performed at the Hirszfled’s Institute of Immunology and Experimental Therapy [397]. A preterm newborn delivered via C-section was hospitalized from the first day of life due to cardiopulmonary failure associated with sepsis. Moreover, the infant developed a neonatal meningitis caused by a *K. pneumoniae* strain susceptible only to imipenem and chloramphenicol. Nevertheless, a treatment with aforementioned antibiotics was ineffective, and the patient was subsequently subjected to the phage therapy using the KL9 bacteriophage. The phage was administered orally for 5 weeks, resulting in complete eradication of the pathogen from cerebrospinal fluid and marked improvement of the child’s health. After 60 h, an infection with *P. aeruginosa* occurred; however, the patient was successfully treated with ceftazidime, to which the bacterial strain was susceptible. Following a puncture of the lateral ventricle, additional complications arose (hemorrhage into central nervous system and internal hydrocephalus), and the boy was transferred to another hospital for further surgical treatment after five months of hospitalization and neurosurgery consultation. This case shows the potential value of bacteriophages in situations where conventional antimicrobial therapy fails. Successful elimination of *K. pneumoniae* from the cerebrospinal fluid suggests that phages may represent an important alternative for managing severe neonatal infections caused by MDR pathogens. Importantly, clinical improvement was achieved despite the patient’s critical condition, highlighting the potential utility of phage therapy in life-threatening neonatal infections. Interestingly, despite the successful eradication of *K. pneumoniae*, the patient subsequently developed a *P. aeruginosa* infection, emphasizing the highly specific nature of bacteriophages and their ability to selectively target a particular pathogen without providing broad antimicrobial coverage.

Furthermore, another treatment was described in 2021, rescuing a 1-year-old child infected with *Enterococcus faecium* [398]. The patient suffered from biliary atresia. She initially underwent portoenterostomy at the age of 8 weeks, which led to severe liver cirrhosis and multiple liver abscesses after recurrent cholangitis. Subsequently, the patient underwent a first liver transplant, which resulted in an infection caused by vancomycin-resistant *E. faecium*. Antibiotic treatment stabilized her vital functions, but even after repetitive abdominal lavages and a revision of the biliodigestive anastomosis, the infection had spread into necrotic aeras. This was associated with ischemia-reperfusion-induced liver injury with delayed graft function, characterized by critically impaired oxygen supply at the microvascular level after transplantation. After the third liver transplant, *E. faecium* was still detected in the abdomen, and the patient was subjected to phage therapy using a cocktail composed of two lytic bacteriophages (EFgrKN and EFgrNG). The cocktail was administered intravenously over two hours, twice daily. During and after the treatment, no adverse effects of bacteriophage administration were noticed. Following the successful phage therapy, the patient experienced a recurrence of infection caused by *K. pneumoniae* and *E. cloacae*; however, these pathogens were also successfully eliminated with antibiotic treatment. Importantly, at 4, 28 and 49 days from the beginning of phage application, no phage-specific antibodies were detected in patient serum samples, indicating that these bacteriophages did not elicit a strong immune response in her body. This case also highlights the ability of bacteriophages to selectively eliminate MDR pathogens while maintaining a favorable safety profile. Interestingly, no phage-specific antibodies were detected despite repeated intravenous administration. As humoral responses against phages are generally expected following systemic exposure [302,303], this observation may reflect the influence of immunosuppressive therapy and underscores the complexity of phage-host immune interactions.

Another case of a full recovery in a child was described by Morozova et al., in which a 3-month-old girl was cured of bronchitis caused by MDR *P. aeruginosa* [399]. The phage cocktail (“Pyobacteriophage”) consisted of two bacteriophages, and was administered for six days followed by five days of treatment with cefotaxime. Phages were applied topically through nasal drops and inhalation. Interestingly, phage treatment altered the antimicrobial profile of this strain, leading to easier antibiotic therapy. This scenario highlighted the potential benefits of combining bacteriophages with conventional antibiotics in the treatment of infections caused by MDR pathogens. Interestingly, phage exposure altered the antimicrobial susceptibility profile of *P. aeruginosa*, facilitating subsequent antibiotic therapy. It suggests that phage therapy can be conducted not solely to eradicate pathogenic bacteria but also to modulate their antibiotic susceptibility.

In November 2025, the first successful treatment of a female infant with pulmonary infection caused by carbapenem-resistant *A. baumannii* and carbapenem-resistant *K. pneumoniae* was recorded [400]. After unsuccessful attempts to eradicate of these pathogens via antibiotic treatment (meropenem, levofloxacin, and polymyxin), a 9-month-old patient was treated with phage therapy. The phage cocktail (composed of the *A. baumannii* specific phage phiAb35 and the *K. pneumoniae* specific phages phiKp240 and phiKP67) was administered using a vibrating-mesh nebulizer; the bacteria were eliminated not only from the lungs but also from the gut after 3 days of treatment, with no adverse effects observed. It demonstrated not only the safe profile of the use of bacteriophages as an antimicrobial alternative but also an opportunity to eradicate two MDR pathogens simultaneously. Since polymicrobial infections pose an enormous threat, these findings presented phage therapy as an important alternative for antibiotics.

An interesting study was conducted in the Pediatric Intensive Care Unit of Boo Ali Sina Hospital in Iraq, where a double-blind clinical trial was performed to evaluate whether a phage cocktail may prevent VAP [401]. Sixty patients (under 18 years of age) were enrolled in this study, with one group (30 patients) receiving placebo and the other group (30 patients) receiving a phage cocktail consisting of phages against *P. aeruginosa* (ATCC No. 27853), *A. baumannii* (ATCC No. BAA-1605), and methicillin-resistant *S. aureus* (ATCC No. 33591). VAP occurred less frequently in a group receiving the phage cocktail (23.4%) than in the placebo group (53.4%). These results may suggest the potential of bacteriophages as a preventive strategy in high-risk pediatric patients. However, routine prophylactic administration should be approached with caution, as large-scale phage exposure may influence the microbial ecology of both patients and hospital environments. Furthermore, only strictly lytic bacteriophages should be considered for such applications. As mentioned, temperate phages may contribute to horizontal gene transfer, spreading bacterial genes within bacterial communities [351]. Future studies should therefore evaluate not only efficacy but also the long-term ecological consequences of preventive phage use.

As mentioned, neonatal sepsis is also one of the major threats to newborns [325]; a cocktail to treat possible sepsis was also proposed [402]. Six bacterial pathogens were selected: *E. coli* (10 isolates), *K. pneumoniae* (10 isolates), *H. influenzae* (four isolates), *P. aeruginosa* (three isolates), *C. freundii* (one isolate) and *M. catarrhalis* (one isolate) to evaluate possible phage cocktails. The cocktail consisted of 29 phages and exhibited lytic activity against 100% of tested bacterial strains. Although it appears highly promising, several important considerations should be taken into account. The implementation of such extensive phage formulations should be approached with caution. Increasing the number of phages may improve pathogen coverage, but it also increases the biological complexity of the preparation and may complicate the prediction of phage–bacteria interactions. Furthermore, some bacteriophages can infect other bacteria than their host species (polyvalent phages [347]), which, in the case of this cocktail can result in alterations in gut microbiota. Therefore, careful characterization of each component, particularly with regard to strictly lytic activity and polyvalence, is vital before clinical application.

Furthermore, in vivo experiments involving the treatment of neonate model organisms were performed. For instance, a bacteriophage Eco30 (targeting *E. coli*) isolated from the feces of a healthy piglet was administered orally to two-day-old piglets in the titre, reaching 10^10^ PFU/mL, providing data in terms of a high-dose phage therapy [403]. Importantly, no single animal suffered from side effects of this therapy. Furthermore, phages supposedly did not pass through the small intestine and assimilated in the colon, which enhanced the information regarding to the activity of bacteriophages within immature organisms. Another study regarding oral phage therapy in newborn piglets was conducted by Wu et al., involving twenty-seven one-day-old piglets infected with *C. perfringens* type C (CVCC1155; host bacteria for vB_CpeP_15N3 phage) [404]. The authors demonstrated that phage administration significantly alleviated clinical symptoms, reduced bacterial load and toxin levels, and improved survival, with particularly strong effects observed in the prophylactic group. Importantly, phage therapy also preserved intestinal integrity and promoted a healthier gut microbiota compared to antibiotic treatment. These findings suggest that prophylactic phage application may represent an especially effective strategy for controlling *C. perfringens* infections in neonatal models [404]. Moreover, it suggests that, rather than complete eradication of all microorganisms, phage therapy may facilitate a selective modulation of pathogenic populations while maintaining the stability of microbiota. It may be critically valuable in terms of neonatal patients, where disruption of early microbial colonization results in long-term consequences.

Although presented case studies and in vivo experiments may serve as a crucial source of empirical data regarding phage therapy protecting newborns from bacterial infections, more research and/or successful treatments are needed in order to define phage therapy as being a promising substitute and/or adjuvant to antibiotic treatment against neonatal MDR infections.

## 6. Other Phage-Based Strategies to Aid Newborns

**Figure 3:**
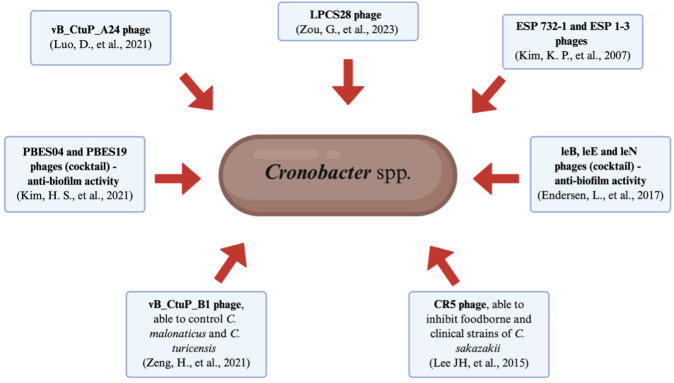
Phages acting as antimicrobial agents against *Cronobacter* spp. [410,411,412,413,414,415,416]. Created in BioRender. https://BioRender.com/fj9f0mn.

Phages are considered as a potential alternative to standard disinfectants due to disinfectant tolerance exhibited by some pathogens, such as *A. baumannii* [405,406]. In some NICUs, it is also impossible to perform chemical disinfection sufficient to fully eradicate pathogens from the environment. *S. capitis* is a dangerous pathogen infecting neonates, which may form biofilms in incubators [407]. It has been reported to exhibit not only resistance to commonly used antibiotics (including vancomycin or aminoglycosides) but also disinfectants, while remaining susceptible to phage cocktails in both planktonic and biofilm forms. Bacteriophages are considered to be a potential disinfectant for dry powder infant formula, which is frequently contaminated by the opportunistic pathogen *Cronobacter sakazakii* [408]. Infection caused by this bacterium can lead to necrotizing colitis and meningitis, with mortality rates reaching up to 80% [409]. *C. sakazakii* has been reported to be resistant to many antibiotics (e.g., tygecycline, cephalosporins) and exhibits high desiccation tolerance, defining it as an enormous threat to infants. Recently, phages have been proposed to act as a possible biocontrol agent against *C. sakazakii* in dry powdered infant formula, and considerable research has been conducted in this field. For instance, the SG01 phage combined with collagen peptide/trehalose-based powders has been reported to exhibit an antibacterial activity against *C. sakazakii* in powdered infant formula [409]. Other phages acting as antimicrobial agents in terms of this pathogen are presented in Figure 3.

Recently, a PhageDx™*Cronobacter* Assay was developed, which can be used for the identification of *Cronobacter* spp. in powder infant formula [417]. The assay relies on the infection of *Cronobacter* cells by specific bacteriophages and the subsequent expression of a luciferase reporter gene. Moreover, phage-derived endolysins are emerging as increasingly important antimicrobial agents [418]. Interestingly, an endolysin derived from JBA6 phage infecting *Bacillus amyloliquefaciens* was shown not only to lyse Gram-negative pathogens such as *E. coli* but also *C.* *sakazakii* [419]. Nevertheless, a novel endolysin, PlyAZ3aT, failed to cure pneumococcal meningitis in an infant rat model caused by a ceftriaxone-resistant clinical strain of *S. pneumoniae* [420].

It is important to mention that the implementation of these phage-based approaches can also be influenced by geographical differences in pathogen prevalence and antimicrobial resistance patterns. While some pathogens, such as *A. baumannii*, represent a major challenge in many low- and middle-income countries and during certain NICU outbreaks, other bacterial species may dominate in different healthcare settings, e.g., *Streptococcus* spp. [236,244]. Hence, the selection of phages for decontamination, prophylaxis, or therapeutic applications will require adaptation to local epidemiological conditions.

## 7. Conclusions

Phage therapy applied to neonatal infections represents a promising therapeutic strategy but needs more investigation and clinical development. Although several case studies have demonstrated the successful application of phage therapy in critically ill newborns, current evidence remains limited. Moreover, despite the significant potential of bacteriophages for *Cronobacter* biocontrol in powdered infant formula, further comprehensive research is still required to elucidate the complex interactions between bacteriophages and the neonatal immune system. Understanding these immunological effects is crucial for accurately predicting potential adverse reactions and ensuring the safety of therapeutic applications in this vulnerable population. Nevertheless, as highlighted by existing case reports, in certain life-threatening infections where conventional treatments fail, phage therapy may represent the most viable and life-saving option. Overall, the available data indicate the potential of the phage therapy in terms of the treatment of newborns suffering from infections caused by MDR pathogens. Nonetheless, this antimicrobial alternative requires more safety establishments, standardized protocols and a deeper understanding of a neonatal immune system response to acquire a robust reliability.

## Figures and Tables

**Table 1:** Microbes involved in the formation of the infant microbiota following vaginal delivery.

Genus	Role in the Infant Microbiota	Developmental Stage of Relevance	Adverse Impact/Pathogenicity	Reference
*Prevotella* spp.	Natural gut commensal involved in vitamin biosynthesis, immune boosting, and carbon metabolism	Initial colonization	Associated with meningitis, dacryocystis, chorioamnionitis (which may lead to sepsis), behavioral problems, pediatric asthma; may be a cause of preterm birth (e.g., by premature rupture of membranes) affecting newborn, e.g., by causing bronchopulmonary dysplasia; correlated with viral bronchiolitis and microbial invasion of the amniotic cavity	[26,27,28,29,30,31,32,33,34,35,36,37,38]
*Lactobacillus* spp.	Probiotic bacteria competing with pathogens (e.g., Group B *Streptococcus*, *Shigella sonnei*, *Salmonella* typhimurium, *S. aureus*, *Clostridium difficile*, and fungi); involved in inhibition of colorectal cancer development and down-regulation of pro-inflammatory cytokines, its presence is correlated with lower possibility of infant colic and necrotizing enterocolitis (NEC)	Initial colonization/ early microbiota development	May be a cause of neurological problems: poor language skills, behavioral problems, lower recognition memory response; very rare cases of bacteremia (usually within infants suffering from a concurrent gastrointestinal complications)	[28,35,39,40,41,42,43,44,45,46,47,48,49]
*Sneathia* spp.	Responsible for shaping the development immune system of a newborn	Initial colonization	Associated with chorioamnionitis, which may lead to sepsis; strongly associated with preterm birth; correlated with microbial invasion of the amniotic cavity	[26,28,50,51,52,53,54,55]
*Atopobium* spp.	An important component of the intestinal microbiota at 3 years of age	Initial colonization/ early microbiota development	Mostly correlated with preterm birth; can induce proinflammatory response within maternal organism affecting her microbiota, hence affecting the newborn’s microbiota and newborn’s neurodevelopment; associated with dental caries or the noma (cancrum oris) during children’s first years of life	[56,57,58,59,60,61,62,63,64,65]
*Clostridium* spp.	Significantly affects the composition of neonate gut microbiota	Early microbiota development	Associated with anaerobic osteomyelitis and bacteremia; *Clostridium neonatale* is linked with NEC and pre-school age asthma; *Clostridium difficile* can be correlated with pseudomembranous colitis, diarrhea, and food allergy considering young children	[49,66,67,68,69,70,71,72,73]
*Bacteroides* spp.	Competing with pathogens causing, e.g., child’s asthma; responsible for human milk oligosaccharides (HMOs) digestion; producer of propionate, which affects inflammation, secretion of cytokines (both pro-inflammatory and anti-inflammatory and microbial ecology; member of the “core microbiota” group of microorganisms; shaping the infant’s immune system, e.g., by stimulation of T-cell production and differentiation	Early microbiota development	Linked with pneumonia, cystis, abscess, conjunctivitis, empyema, bacteremia, meningitis, leukemia, rectal abscess, perforated appendix, prematurity, NEC, empyema, and immune deficiency; associated with neurological problems: poor language skills, behavioral problems, sub-dimensions frustration, sadness, and lower negative affectivity; may be associated with gestational diabetes, mellitus exposure and gut microbiota immaturity; linked with infantile colic	[35,49,63,74,75,76,77,78,79,80,65,81,82,83,84]
*Phocaeicola* spp.	One of the first microorganisms colonizing infant’s gut; commensal competing with pathogens (e.g., *E. coli*); correlated with higher abundance of bacteriophages within infant’s gut; degradation of plant-derived heteropolysaccharides; a keystone of infant’s microbiota affecting many metabolic pathways	Initial colonization	Low colonization by these species may affect cognitive performances (e.g., reduced memory functions); reduced abundance of these species was linked with anterior uveitis	[22,85,86,87]
*E. coli*	Predominant commensal inside gastrointestinal tract competing with other pathogens; responsible for production of β-glucuronidase and enhancement of enterohepatic circulation in the gut; responsible for production of vitamin K and B12; by consuming oxygen may help growing other commensal microorganisms crucial for the gastrointestinal tract equilibrium; may reduce the colonization of *Salmonella typhimurium* within the gut	Initial colonization/ early microbiota development	Most common bacterial pathogen affecting neonates; associated with neonatal meningitidis, diarrhea, neonatal sepsis, urinary tract infection (UTI), pyelonephritis, bacteremia, jaundice, acute focal bacterial nephritis, mediastinitis, cerebellar stroke, hemolytic uremic syndrome, bloodstream infections, NEC, pregnancy-associated asymptomatic bacteriuria	[49,88,89,90,91,92,93,94,95,96,97,98,99,100,101,102,103,104,105,106]

**Table 2:** Microbes involved in forming an infant microbiota following C-section delivery.

Genus	Role in Infant Microbiota	Developmental Stage of Relevance	Adverse Impact/Pathogenicity	Reference
*Corynebacterium* spp.	As a commensal competing with other pathogens within hypopharyngeal and nasopharyngeal microbiota (e.g., *Haemophilus* sp.) may decrease asthma risk; competing with *Streptococcus pneumoniae* within nasopharyngeal microbiota preventing from infections (which may lead to e.g., otitis media or bronchiolitis)	Early microbiota development	Can be linked with endocarditis; *Corynevbacterium kroppenstedtii* and *Corynebacterium diphteriae* may be associated with bloodstream infections; *Corynebacterium aurimucosum* may cause neonatal septic meningitis; *Corynebacterium pseudodiphtheriticum* may cause exudative pharyngitis; *Corynebacterium coyleae* was correlated with UTI; before obligatory vaccination, *C. diptheriae* was associated with diphteria	[107,108,109,110,111,112,113,114,115,116,117,118,119,120,121]
*Staphylococcus* spp.	*Staphylococcus epidermidis* is an important commensal component of skin microbiota Enhancing skin barrier; an important bacterium involved in maintaining middle ear microbiota	Initial colonization/ early microbiota development	Can be associated with cystic fibrosis, developing food allergy, neonatal sepsis, endocarditis, neonatal conjunctivitis osteoarticular infections (e.g., osteomyelitis), bacteremia, NEC, late-onset neonatal sepsis (which may lead to alteration in neurodevelopment), pyomyositis, pregnancy-associated asymptomatic bacteriuria, scaled skin syndrome, ecthyma gangrenosum, UTI; was correlated with recurrent fatal pyopneumothorax or empyema thoracic (which were caused by pneumonia or SARS-CoV2 respectively)	[49,68,90,92,97,105,122,123,124,125,126,127,128,129,130,131,132,133,134,135,136,137,138,139,140,141,142]
*Propionibacterium* spp.	Plays an important role in skin homeostasis; present in healthy individuals, with regard to those affected by NEC, which suggests inhibition of pathogens causing this disease; plays an important role in the development of inflammation (induces the differentiation of bacteria-specific Th17 lymphocytes); competing with pathogens forming a skin barrier (e.g., against *S. aureus*); an important bacterium involved in maintaining middle ear microbiota	Early microbiota development	Associated with endodontic infections, infectious pericarditis and bacteremia; may cause immunostimulation	[67,123,143,144,145,146,147]
*Klebsiella* spp.	Commensal (saprophyte) in gastrointestinal and respiratory tract; *Klebsiella michiganensis* may impede gut colonization for *E. coli*	Initial colonization/ early microbiota development	Associated with sepsis, necrotizing small bowel colitis, neonatal meningitis, osteoarticular infections, neonatal and late-onset sepsis, pregnancy-associated asymptomatic bacteriuria, UTI, NEC, bacteremia (which may lead to tachycardia, fever or apnea episodes)	[39,49,90,92,97,101,140,148,149,150,151,152,153,154,155,156,157]
*Enterobacter* spp.	*Enterobacter ludwigii* possibly protects from colitis	Initial colonization/ early microbiota development	Can be correlated with osteoarticular infections, neonatal and late-onset sepsis, bacteremia, meningitis, NEC, UTI	[49,122,140,152,155,158,159,160,161]
*Clostridium* spp.	Significantly affects the composition of neonate gut microbiota	Early microbiota development	Associated with anaerobic osteomyelitis and bacteremia; *C. neonatale* is linked with NEC and pre-school age asthma; *C. difficile* can be correlated with pseudomembranous colitis, diarrhea, and food allergy considering young children	[49,66,67,68,69,70,71,72,73]
*Veillonella* spp.	Maintaining acidic pH as a protectant against other pathogen’s biofilms; present in healthy individuals, with regard to those affected by NEC, which suggests inhibition of pathogens causing this disease; involved in the production of short-chain fatty acids	Early microbiota development	May be associated with gestational diabetes mellitus exposure, gut microbiota immaturity, infections, and bacteremia; correlated with the immunopathology of cholestasis and meningitis; may cause asthma at the age of 6; *V. parvula* was correlated with causing pyogenic intraventricular empyema	[18,49,67,84,147,162,163,164,165,166,167]

**Table 3:** Microbes involved in forming an infant microbiota composition characteristic for natural breastfeeding of an infant.

Genus	Role in Infant’s Microbiota	Developmental Stage of Relevance	Adverse Impact/Pathogenicity	Reference
*Staphylococcus* spp.	*S. epidermidis* is an important commensal within skin microbiota enhancing skin barrier; an important bacterium involved in maintaining middle ear microbiota	Initial colonization/ early microbiota development	Can be associated with cystic fibrosis, developing food allergy, neonatal sepsis, neonatal conjunctivitis, endocarditis, osteoarticular infections (e.g., osteomyelitis), bacteremia, NEC, late-onset neonatal sepsis (which may lead to alteration in neurodevelopment), pyomyositis, pregnancy-associated asymptomatic bacteriuria, scaled skin syndrome, ecthyma gangrenosum; infectious pericarditis, UTI; correlated with recurrent fatal pyopneumothorax or empyema thoracic (which were caused by pneumonia or SARS-CoV2 respectively)	[49,68,90,92,97,105,122,123,124,125,126,127,128,129,130,131,132,133,134,135,136,137,138,139,140,141,142]
*Serratia* spp.	Mostly pathogenic	Initial colonization/ early microbiota development	May be a reason of neonatal conjunctivitis, keratitis, lower respiratory tract infections (leading to pneumonia), bacteremia, neonatal sepsis, intracranial and intestinal infections, UTI, meningitis, endocarditis; mother affected by this bacterium might suffer from chorioamnionitis	[39,49,142,185,186,187,188,189,190]
*Streptococcus* spp.	One of the most prevalent bacteria in breastmilk; *Streptococcus agalactiae* or Group B *Streptococcus* are commensals in gastrointestinal and genitourinary tract, and oropharynx; metabolic products derived from reactions (maintained by *Streptococcus* sp.) involving oligosaccharides present in breast milk as substrates, allow a colonization of other bacteria (e.g., *Actinomyces* sp.); an important bacterium involved in maintaining middle ear microbiota	Initial colonization	May be a reason of bacteremia, early-onset neonatal disease (EOD), neonatal sepsis, neonatal parotitis, meningitis, pericarditis, NEC, pneumonia, impaired neurodevelopment (e.g., learning disabilities, autistic and cerebral palsy traits), osteomyelitis, tooth decay; mother affected by this bacterium might suffer from chorioamnionitis; might cause preterm birth and stillbirth	[49,123,191,192,193,194,195,196,197,198,199,200,201]
*Pseudomonas* spp.	Mostly pathogenic	Initial colonization	May be correlated with ecthyma gangrenosum, peritonitis, meningitis, UTI, cystic fibrosis, neonatal sepsis, bloodstream infections, conjunctivitis, keratitis, subcutaneous nodules and mastoid bone destruction	[39,49,128,202,203,204,205,206,207,208,209,210]
*Corynebacterium* spp.	A commensal competing with other pathogens within hypopharyngeal and nasopharyngeal microbiota (e.g., *Haemophilus* sp.) may decrease asthma risk; competing with *Streptococcus pneumoniae* within nasopharyngeal microbiota preventing from infections (which may lead to, e.g., otitis media or bronchiolitis)	Early microbiota development	Can be linked with endocarditis; *C. kroppenstedtii* and *C. diphteriae* may be associated with bloodstream infections; *C. aurimucosum* may cause neonatal septic meningitis; *C. pseudodiphtheriticum* may cause exudative pharyngitis; *C. coyleae* was correlated with UTI; before obligatory vaccination, *C. diptheriae* was associated with diphteria	[107,108,109,110,111,112,113,114,115,116,117,118,119,120,121]
*Ralstonia* spp.	Maintaining proper newborn birth weight; involved in maintaining middle ear microbiota	Initial colonization/ early microbiota development	Associated with UTI, bacteremia, neonatal sepsis, NEC, meningitis, septicemia	[123,211,212,213,214,215,216,217]
*Propionibacterium* spp.	Has an important role in skin homeostasis; present in healthy individuals, with regard to those affected by NEC, which suggests inhibition of pathogens causing this disease; plays an important role in the development of inflammation (induces the differentiation of bacteria-specific Th17 lymphocytes); competing with pathogens forming a skin barrier (e.g., against *S. aureus*); an important bacterium involved in maintaining middle ear microbiota	Early microbiota development	Associated with endodontic infections, infectious pericarditis and bacteremia; may cause immunostimulation	[67,123,143,144,145,146,147]
*Sphingomonas* spp.	Mostly acting as opportunistic pathogens	Early microbiota development	Correlated with food sensitization, bronchiolitis, systemic lupus erythematosus, meningitis, bacteremia, endocarditis, soft tissue infection, UTI, infections within the central nervous system, septic arthritis, pneumonia, septicemia, biliary tract infection, wheezing; *Sphingomonas paucimobilis* and *Sphingomonas echinoides* are known to be opportunistic pathogens for the newborns amongst *Sphingomonas* sp.	[178,218,219,220,221,222,223]
*Bifidobacterium* spp.	Dominates the infant gut microbiota; balances (anti-inflammatory properties) and accelerates the maturation of the immune system, increases acetate production, improves intestinal barrier function; its presence is correlated with lower possibility of infant colic and NEC, responsible for the assimilation of HMOs and production of short-chain fatty acids (important for cardiovascular health and/or prevention from colorectal tumorigenesis), correlated with the protection against atopic eczema, celiac disease, biliary atresia, and other diseases caused by infections of pathogens with whom *Bifidobacterium* sp. compete as a commensal (by modulating mucosal barrier function and promoting immunological, and inflammatory response); improves the response to vaccination; assures a protection from food sensitization and food allergy	Initial colonization/ early microbiota development	Known as a “good bacterium”, commensal; very rare cases of bacteremia (usually within infants suffering from a concurrent gastrointestinal complications)	[12,39,41,48,49,224,225,40,226,227,228,229,230,231]
*Bradyrhizobium* spp.	As a commensal competing with other pathogens within gut microbiota	Initial colonization	Associated with bloodstream infections, early onset neonatal sepsis	[232,233]

**Table 4:** Microbes most commonly correlated with the contaminated human donor milk.

Genus	Role in Infant Gut Microbiota	Developmental Stage of Relevance	Adverse Impact/Pathogenicity	Reference
*Staphylococcus* spp.	*S. epidermidis* is an important commensal within skin microbiota enhancing skin barrier; an important bacterium involved in maintaining middle ear microbiota	Initial colonization/ early microbiota development	Can be associated with cystic fibrosis, developing food allergy, neonatal sepsis, neonatal conjunctivitis, endocarditis, osteoarticular infections (e.g., osteomyelitis), bacteremia, NEC, late-onset neonatal sepsis (which may lead to alteration in neurodevelopment), pyomyositis, pregnancy-associated asymptomatic bacteriuria, scaled skin syndrome, ecthyma gangrenosum; infectious pericarditis, UTI; was correlated with recurrent fatal pyopneumothorax or empyema thoracic (which were caused by pneumonia or SARS-CoV2 respectively)	[49,68,90,92,97,105,122,123,124,125,126,127,128,129,130,131,132,133,134,135,136,137,138,139,140,141,142]
*Bacillus* spp.	Mostly acts as a pathogen in terms of newborns	Early microbiota development	May be associated with infections of the central nervous system, meningitis, empyema; bacteremia, sepsis; respiratory tract infections (pneumonia); skin infections; gastrointestinal infections; osteoarticular infections (arthritis, osteitis); kidney and urinary tract infections	[234,235]
*Acinetobacter* spp.	Mostly acts as a pathogen in terms of newborns	Initial colonization/ early microbiota development	One of the most causative agents of respiratory tract infections (e.g., ventilator associated pneumonia (VAP), early-onset sepsis (which may be following septic arthritis); correlated with feeding intolerance in preterm infants; meningitis (possibly following symptomatic subdural hygroma, neonatal pneumocephalus); neonatal conjunctivitis; suppurative parotitis; neonatal purpura fulminans; NEC; infective endocarditis	[49,213,236,237,238,239,240,241,242,243,244,245,246,247]
*Micrococcus* spp.	Its lower abundance in nasopharyngeal microbiota is correlated with a possibility for acute otitis media, which might stand for the fact that it is a commensal competing with pathogenic bacteria; natural commensal, shaping skin microflora	Early microbiota development	Known as non-pathogenic bacterium; can be associated with bacteremia (leading to bloodstream infection and/or sepsis)	[248,249,250,251]
*Corynebacterium* spp.	As a commensal competing with other pathogens within hypopharyngeal and nasopharyngeal microbiota (e.g., *Haemophilus* sp.) may decrease asthma risk; competing with S. pneumoniae within nasopharyngeal microbiota preventing from infections (which may lead to, e.g., otitis media or bronchiolitis)	Early microbiota development	Can be linked with endocarditis; *C. kroppenstedtii* and C. *diphteriae* may be associated with bloodstream infections; *C. aurimucosum* may cause neonatal septic meningitis; *C. pseudodiphtheriticum* may cause exudative pharyngitis; *C. coyleae* was correlated with UTI; before obligatory vaccination, C. diptheriae was associated with diphteria	[107,108,109,110,111,112,113,114,115,116,117,118,119,120,121]
*Propionibacterium* spp.	Has an important role in skin homeostasis; present in healthy individuals, with regard to those affected by NEC, which suggests inhibition of pathogens causing this disease; plays an important role in the development of inflammation (induces the differentiation of bacteria-specific Th17 lymphocytes); competing with pathogens forming a skin barrier (e.g., against *S. aureus*); an important bacterium involved in maintaining middle ear microbiota	Early microbiota development	Associated with endodontic infections, infectious pericarditis and bacteremia; may cause immunostimulation	[67,123,143,144,145,146,147]
*Enterobacter* spp.	*E. ludwigii* possibly protects from colitis	Initial colonization/ early microbiota development	Can be linked with osteoarticular infections, neonatal and late-onset sepsis, bacteremia, meningitis, NEC, UTI	[49,122,140,152,155,158,159,160,161]
*Klebsiella* spp.	Commensal (saprophyte) in gastrointestinal and respiratory tract; *K. michiganensis* may impede gut colonization for *E. coli*	Initial colonization/ early microbiota development	Associated with sepsis, necrotizing small bowel colitis, neonatal meningitis, osteoarticular infections, neonatal and late-onset sepsis, pregnancy-associated asymptomatic bacteriuria, UTI, NEC, bacteremia (which may lead to tachycardia, fever or apnea episodes)	[39,49,90,92,97,101,140,148,149,150,151,152,153,154,155,156,157]
*E. coli*	Predominant commensal inside gastrointestinal tract competing with other pathogens; responsible for production of β-glucuronidase and enhancement of enterohepatic circulation in the gut; responsible for production of vitamin K and B12; by consuming oxygen may help growing other commensal microorganisms crucial for the gastrointestinal tract equilibrium; may reduce the colonization of *S. typhimurium* within the gut	Initial colonization/ early microbiota development	Most common bacterial pathogen affecting neonates; associated with neonatal meningitidis, diarrhea, neonatal sepsis, UTI, pyelonephritis, bacteremia, jaundice, acute focal bacterial nephritis, mediastinitis, cerebellar stroke, hemolytic uremic syndrome, bloodstream infections, NEC, pregnancy-associated asymptomatic bacteriuria	[49,88,89,90,91,92,93,94,95,96,97,98,99,100,101,102,103,104,105,106,107]
*Neisseria* spp.	Mostly pathogenic; might prevent dental caries and periodontal disease and competes with pathogenic *Fusobacterium nucleatum; Neisseria lactamica* is known to be a natural pharyngeal commensal	Early microbiota development	Causative agent of an invasive meningococcal disease (leading to pneumonia, arthritis, otitis media, epiglottitis, and encephalitis); correlated with meningococcal eye infection, childhood bacteremia (leading to sepsis), conjunctivitis (ophthalmia neonatorum); associated with respiratory tract infection; *Neisseria oralis* may cause septicemia	[252,253,254,255,256,257,258,259,260,261]
*Serratia* spp.	Mostly acts as a pathogen in terms of newborns	Initial colonization/ early microbiota development	May be a reason of neonatal conjunctivitis, keratitis, lower respiratory tract infections (leading to pneumonia), bacteremia, neonatal sepsis, intracranial and intestinal infections, UTI, meningitis, endocarditis; mother affected by this bacterium might suffer from chorioamnionitis	[39,49,142,185,186,187,188,189,190]
*Enterococcus* spp.	Phages associated with *Enterococcus* spp. have been shown to enhance T-cell immunity, and certain strains of *Enterococcus faecalis* have demonstrated the ability to counteract NEC pathology	Initial colonization/ early microbiota development	Associated with biliary atresia; bloodstream infections (possibly leading to sepsis); meningitis; respiratory tract infections; febrile urinary tract infections; NEC; correlated with acute focal bacterial nephritis	[49,99,227,262,263,264,265,266,267,268,269]
*Streptococcus* spp.	One of the most prevalent bacteria in breastmilk; *S. agalactiae* or Group B *Streptococcus* are commensals in gastrointestinal and genitourinary tract, and oropharynx; metabolic products derived from reactions (maintained by *Streptococcus* sp.) involving oligosaccharides present in breast milk as substrates, allow a colonization of other bacteria (e.g., *Actinomyces* sp.); an important bacterium involved in maintaining middle ear microbiota	Initial colonization	May be a reason of bacteremia, EOD, neonatal sepsis, neonatal parotitis, meningitis, pericarditis, NEC, pneumonia, impaired neurodevelopment (e.g., learning disabilities, autistic and cerebral palsy traits), osteomyelitis, tooth decay; mother affected by this bacterium might suffer from chorioamnionitis; might cause preterm birth and stillbirth	[49,123,191,192,193,194,195,196,197,198,199,200,201]

## Data Availability

Not applicable.

## Abbreviations

C-section	Caesarean section
CrAssphage	Cross-assembly phage
dsDNA	Double-stranded DNA
EOD	Early-onset neonatal disease
ESKAPE	*Enterococcus* spp.; *Staphylococcus aureus*; *Klebsiella pneumoniae*; *Acinetobacter baumannii*; *Pseudomonas aeruginosa*; *Enterobacter* spp.
HMOs	Human milk oligosaccharides
MDR	Multidrug resistant
MRSA	Methicillin-resistant *Staphylococcus aureus*
NEC	Necrotizing enterocolitis
NICU	Neonatal intensive care unit
RBP	Receptor binding protein
UTI	Urinary tract infection
VAP	Ventilator-associated pneumonia
WHO	World Health Organization
